# Graphene/silver nanoflower hybrid coating for improved cycle performance of thermally-operated soft actuators

**DOI:** 10.1038/s41598-020-74641-5

**Published:** 2020-10-16

**Authors:** Chengxu Piao, Ji Won Suk

**Affiliations:** 1 School of Mechanical Engineering, Sungkyunkwan University, Suwon, Gyeonggi-do 16419 Republic of Korea; 2 SKKU Advanced Institute of Nanotechnology (SAINT), Sungkyunkwan University, Suwon, Gyeonggi-do 16419 Republic of Korea

**Keywords:** Mechanical engineering, Nanoscale materials, Graphene, Nanoscale materials

## Abstract

Twisted and coiled actuators (TCAs), fabricated by twisting cheap nylon sewing threads, have attracted a great deal of attention for their use as artificial muscles or soft actuators. Since the dynamic behavior of a thermally-operated TCA is governed by its thermal properties, graphene and silver nanoflowers (AgNFs) were spray-coated onto the surface of an actuator to achieve enhanced heat transfer. Addition of AgNFs improves interfacial thermal contacts between graphene flakes, while pristine graphene flakes have extremely high in-plane thermal conductivity. Thus, the synergistic effect of graphene and AgNFs reduced the total cycle time of the TCA by up to 38%. Furthermore, when a pulsed current with a 40% duty cycle was applied to the TCA, the graphene/AgNF-coated TCA exhibited a threefold larger peak-to-peak amplitude of the displacement oscillation of the actuator, as compared to that of the non-coated TCA, which demonstrates that the combination of graphene and AgNFs effectively reduced a cooling time of the TCA. This work shows great potential for a simple coating of graphene and AgNFs to produce high-performance thermally-operated soft actuators.

## Introduction

Muscle-like actuators or soft actuators are a key component of advanced applications such as robotics, wearables, and medical devices because conventional actuators are bulky, heavy, rigid, and noisy^1–3^. Therefore, a great deal of effort has been invested in developing muscle-like actuators with high mechanical compliance and high specific power, including shape memory alloys (SMAs)^4,5^, pneumatic elastomeric actuators^6^, electro-active polymers (EAPs)^7,8^, twisted and coiled actuators (TCAs)^9,10^, and others. Among these soft actuators, TCAs fabricated by twisting and coiling sewing threads made of inexpensive polymers demonstrated an extremely high specific power of 5.3 kW/kg along with additional advantages including a large operating strain of over 20%, small hysteresis, high flexibility, low weight, and low fabrication cost^9^, which makes them a promising candidate for practical artificial muscles.

When making TCAs, extreme twisting of polymer fibers forms spring-like coils and the gaps between coils are controlled by temperature change of the actuator; when the actuator is heated, the gaps between coils are closed and the actuator contracts. Repeated heating and cooling are typically achieved by applying hot air or flowing hot water through a tube surrounding the TCA^9^. However, this requires additional bulky components for temperature control of the TCA. Thus, conductive silver-coated polymer fibers were used to fabricate TCAs that enable temperature control by application of an electric current through the actuator for Joule heating^11^. This is a simple and efficient method for operating the TCAs with controlled actuation.

The dynamic behaviors of a TCA are dependent on its thermal properties. Since polymer fibers have relatively low thermal conductivity, other materials, such as carbon nanotubes (CNTs), have been utilized to fabricate super-coiled actuators. A coiled yarn made from sheets of CNTs demonstrated fast tensile actuation with large forces and strokes because of the high thermal and electrical properties of CNTs^12^. However, twist-spun CNT yarns require a complicated fabrication process involving spinnable CNT forests, resulting in high fabrication cost and technical difficulties. To take advantage of TCAs made from cheap polymer fibers, a simple coating of graphene-based materials was recently introduced to enhance dynamic performance^13^. High-quality monolayer graphene with minimal defects has shown an extremely high thermal conductivity of over 2000 W/mK^14–16^. Thus, TCAs fabricated from nylon fibers were coated with various forms of graphene, such as monolayer large-area graphene, three-dimensional (3D) graphene foam, and graphene flakes, which enabled a reduction in the cycle time of the TCA; in particular, coating of graphene flakes demonstrated a dramatic decrease in the cycle time by 30.9%^13^. However, it has long been known that the out-of-plane thermal conductivity of graphitic materials is two orders of magnitude lower than the in-plane thermal conductivity due to the weak van der Waals interactions between individual graphene layers^17^ and the weak interaction between graphene flakes increases the interfacial thermal resistance. This limits the enhancement of the thermal properties of the graphene-based coating layer. In this respect, there is still a demand for coating layers comprised of graphene flakes with improved thermal properties that would allow for creation of high-performance TCAs. Metallic nanoparticles have been used to improve the thermal conductivity by bridging two-dimensional (2D) materials^18^. Recently, flower-shaped silver nanoparticles (silver nanoflowers, AgNFs) have been developed for conductive electrodes and composites^19,20^. AgNFs have single-crystal petals that are around 20 nm thick. Due to their hierarchical structure with ultrathin petals, they provide better dispersion without agglomeration and allow for coalescence of petals at low temperatures of 80–120 °C^19^. Therefore, they have great potential for enhancing the interfacial thermal conductance between 2D materials by melting the silver petals and generating improved physical contacts to 2D materials.

In this study, AgNFs were embedded into graphene flakes to enhance thermal contacts between graphene flakes and thus improve the dynamic performance of TCAs. Since low-temperature curing can induce the active coalescence of silver petals, the interfaces between graphene flakes can be bridged by the AgNFs, ensuring better thermal contacts. AgNFs mixed with graphene flakes were spray-coated on the surface of the TCAs. The enhanced dynamic performance of the TCA coated with the graphene/AgNF hybrids was investigated by cyclic operations under repeated heating and cooling.

## Experimental methods

### Fabrication of TCAs coated with graphene/AgNF hybrids

**Figure 1:**
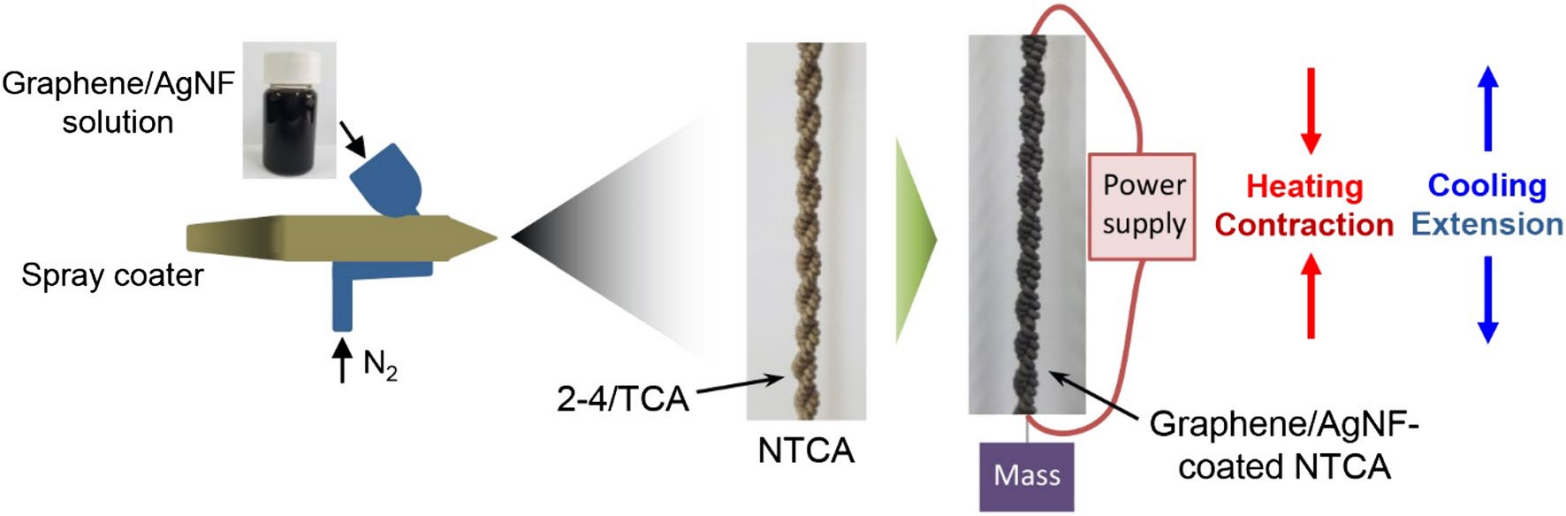
Schematic illustration of coating 2–4/TCAs with graphene/AgNF hybrids and actuation by Joule heating.

TCAs were fabricated using commercial silver-coated nylon fibers (nylon 6, 6 sewing threads, 260151023534, Shieldex). TCA performance is dependent on fabrication method. Following previous works^13,21^, a 2–4/TCA was used to maximize the stroke under an applied load (Fig. 1). Four nylon fibers were first twisted to form one twisted and coiled yarn. A 2–4/TCA was formed by plying two twisted and coiled yarns. Since the fabricated TCAs initially had densely packed coils, the TCAs were exposed to repeated heating and cooling with a 1 kg payload to generate gaps between the coils.

Pristine multi-layer graphene was used as a coating material after exfoliation of graphite nanoplatelets (M-5, XG Sciences) by sonication^22^. A dispersion of graphene flakes (5 mg/mL) was prepared in a water/ethanol mixture with a volume ratio of 1:9^13^. After AgNFs were synthesized using a previously published method^19^, AgNF powder was poured into the graphene dispersion. The fabricated TCAs were spray-coated with the mixture of graphene and AgNFs using a spray gun and dried in air (Fig. 1).

### Characterization of materials

Scanning electron microscopy (SEM, JSM-7600, Jeol) was used to observe graphene flakes, AgNFs, and the surface of the fabricated TCAs. The surface morphology and the cross-sectional view of graphene flakes were observed by transmission electron microscopy (TEM, ARF 200F, Jeol). Raman spectroscopy (ALPHA300M, WiTec) with a 532 nm wavelength laser was employed to characterize the graphene flakes.

### Testing of TCA cycle performance

As the TCAs were fabricated with electrically conductive silver-coated nylon fibers, the dynamic performance of the fabricated actuators was evaluated using Joule heating. A DC current was applied to the two ends of the TCAs to control the temperature for actuation. A detailed experimental set-up was described in previous papers^10,13,21^. Briefly, the temperature of the TCAs was monitored with a micro thermistor placed on the end of the TCAs, which enabled closed-loop temperature control. A linear magnetic encoder was used to measure the displacement of the TCAs under a temperature range between 35 °C and 100 °C, while a 500 g payload was attached to the end of the vertically-positioned TCAs.

## Results and discussion

### TCAs coated with graphene/AgNW hybrids

**Figure 2:**
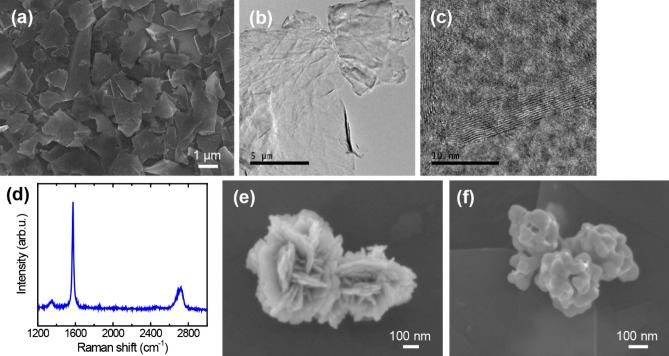
Graphene/AgNF hybrid coating materials. (**a**) SEM image of graphene flakes on Si. (**b**) TEM image of graphene flakes. (**c**) High-resolution TEM image of graphene flakes in cross-section. (**d**) Raman spectrum of graphene flakes. (**e**) SEM image of AgNFs on Si. (**f**) SEM image of AgNFs that coalesced on graphene flakes after heat treatment.

Figure 2a shows exfoliated multi-layer graphene flakes on a silicon substrate. The flakes had lateral dimensions of a few micrometers. A high-resolution TEM (HR-TEM) image depicts 2D nature with an atomic thickness of the exfoliated graphene flakes (Fig. 2b). The cross-sectional view of the flakes by HR-TEM shows clear graphene layers with a total thickness of below 10 nm (Fig. 2c). Raman spectroscopy of the graphene flakes exhibited the G band at 1580 cm^−1^ and the 2D band at 2700 cm^−1^ (Fig. 2d). The lower intensity of the 2D band compared to the G band confirms that the graphene flakes have multiple layers^23,24^, which is in good agreement with the HR-TEM observation. In addition, the D band at around 1350 cm^−1^ is minimal, indicating that the graphene flakes are high in quality with minimal defects^25^.

Figure 2e shows AgNFs on a silicon substrate. The flower-shaped silver particles have several petals that are tens of nanometers thick. The ultrathin petals enable coalescence of AgNFs at low temperatures of 80–120 °C^19^. As shown in Fig. 2e,f, the petals of the AgNFs placed on graphene flakes melted after heat treatment at 100 °C. Owing to the low-temperature coalescence of the AgNFs, they can provide thermal bridges between the graphene flakes without degrading the TCAs. The maximum operating temperature of the TCAs is 100 °C in this study. Thus, heat treatment at 100 °C is acceptable for fabrication of the TCAs.

**Figure 3:**
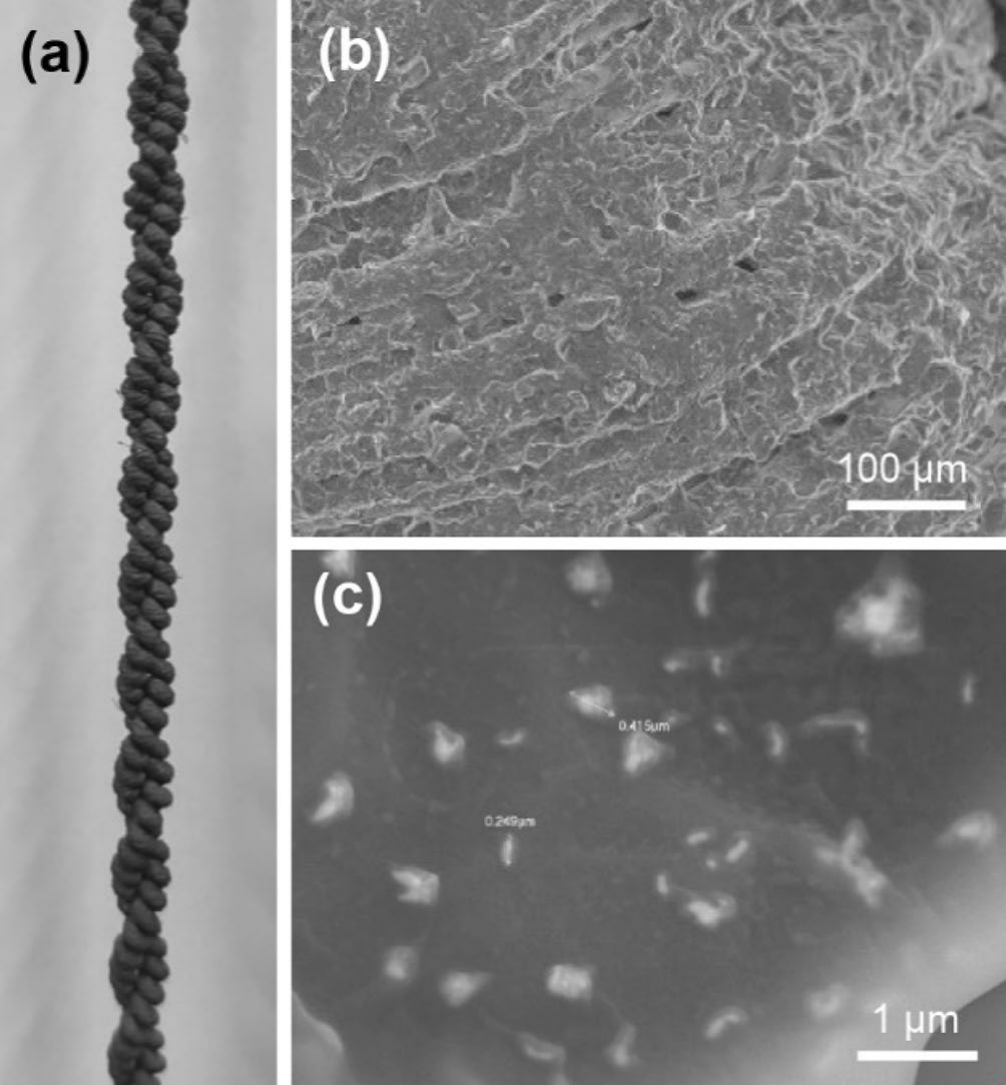
(**a**) A TCA coated with graphene/AgNF hybrids. (**b**) SEM image of the surface of the TCA. (**c**) High-magnification SEM image of the surface of the coating layer.

Figure 3 shows the TCAs coated with graphene/AgNF hybrids. By spray-coating, the whole TCA was uniformly coated with the hybrid (Fig. 3a). Figure 3b confirms that each fiber of the TCAs was covered with graphene and AgNFs. Furthermore, the high-magnification SEM image of the surface of the TCAs shows well-dispersed AgNFs embedded in graphene flakes (Fig. 3c).

### TCA cycle performance

**Figure 4:**
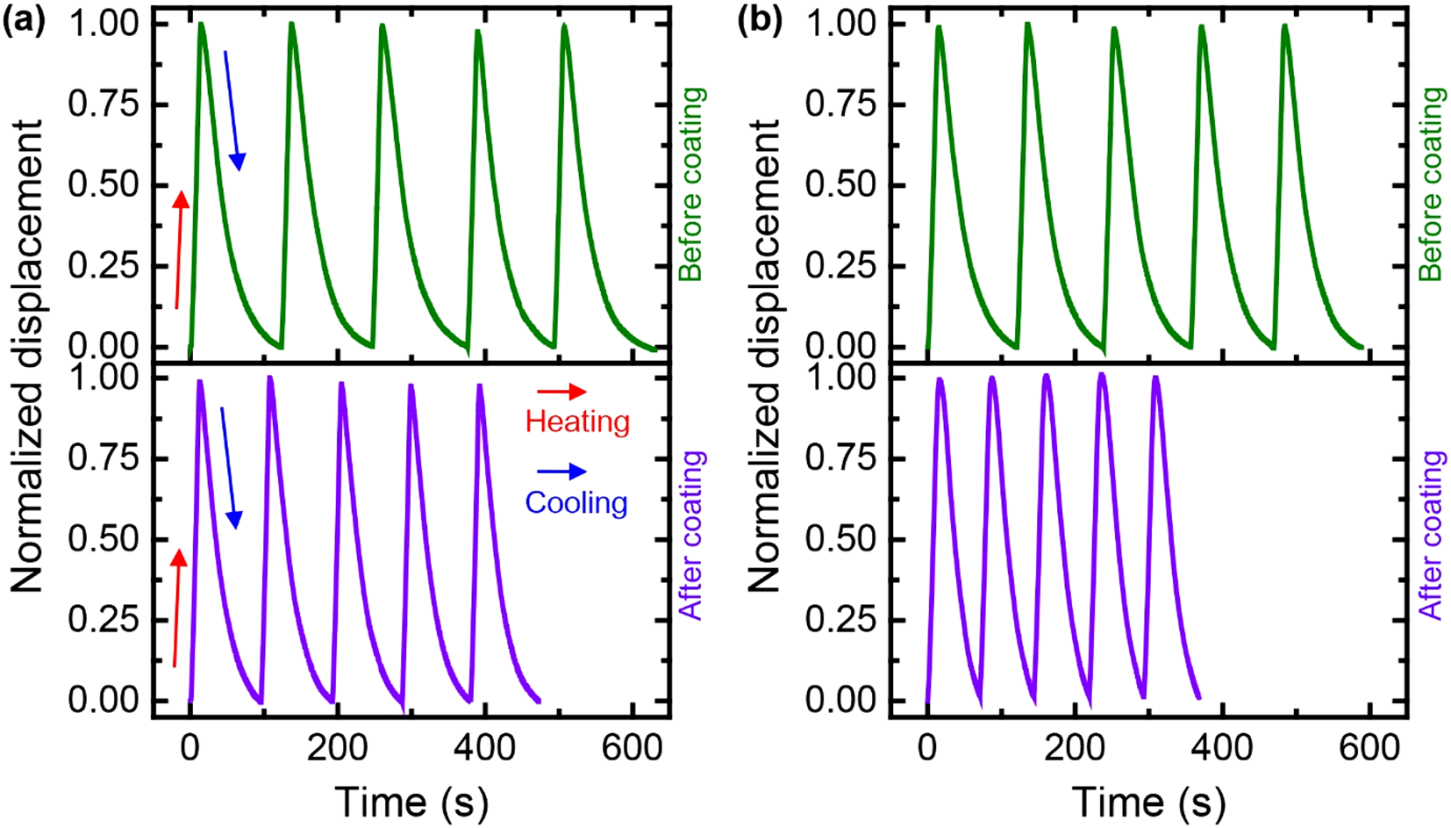
Response to repeated heating and cooling cycles for TCAs coated with (**a**) graphene or (**b**) graphene/AgNF. The mass ratio of AgNFs to graphene was 1 for the actuator shown in (**b**).

TCAs coated with graphene/AgNF hybrids were tested with repeated heating and cooling. Displacements of each TCA were normalized to the maximum displacement in order to compare the cycle performance of different TCAs^13,26^. The following equation expresses the normalized displacement:

$${\text{Normalized }}\;{\text{displacement}} = \frac{d}{{d_{\max } }}$$

where d_max_ is the difference between the length of the TCA at the lowest and highest temperatures and d is the difference between the length of the TCA at the lowest and given temperatures. When the TCA is heated, it contracts and reaches the maximum value (1) of the normalized displacement. When the current is shut off, the TCA starts to naturally cool from exposure to air and extends to its maximum length (minimal normalized displacement, 0) at the lowest operating temperature. Figure 4 depicts the dynamic response of the TCA before and after the coating over the course of 5 cycles (repeated heating and cooling). The coating significantly reduced the cycle time (heating and cooling time for TCA actuation).

A first-order linear model can be used to understand the thermal behavior of a TCA with the following equation^27^:

$$C_{th} \frac{dT(t)}{{dt}} = P(t) - \lambda (T(t) - T_{0} )$$

where T is the temperature of the actuator, T_0_ is the ambient temperature, P is the input electric power, and C_th_ and λ are the thermal mass and thermal conductivity of the thermally-operated actuator, respectively. The dynamic performance of the TCA can be determined by the time constant, τ = C_th_/λ. Therefore, increasing the thermal conductivity of the TCA is one way to improve its cycle performance. As the thermal conductivity of pristine graphene is extremely high, a simple coating of graphene flakes on the surface of the TCA enhances the thermal response of the actuator^13^. However, the out-of-plane thermal conductivity of multi-layer graphene flakes is two orders of magnitude lower than the in-plane thermal conductivity^17^ and the weak interaction between graphene flakes induces thermal resistances at interfaces.

To obtain better thermal interfaces between graphene flakes, AgNFs were embedded into the graphene flakes. Silver nanomaterials with different morphologies have been used to bridge other electrically and thermally conductive nanomaterials. For instance, silver nanowires (AgNWs) provided robust electrical and thermal connections for graphene^28^ and CNTs^29^ due to their high-aspect-ratio geometries and outstanding physical properties. However, our study (not shown here) revealed that the use of AgNWs could not grant the TCAs better dynamic behavior. This may be because long AgNWs reinforced the coating layer, interfering with TCA actuation. In addition, silver nanoparticles (AgNPs) have been utilized synergistically with other nanomaterials^30^. However, AgNPs tend to easily agglomerate^31^, which can increase stiffness of the TCAs. Due to their better dispersibility, high thermal and electrical conductivities, and ability to coalesce at very low temperatures, AgNFs offer thermally robust and mechanically flexible bridges between graphene flakes in the TCA coating.

The synergistic effect of graphene/AgNF hybrids was investigated by evaluating the cycle performance of the coated TCAs. Heating, cooling, and total cycle times were evaluated from the dynamic responses shown in Fig. 4. Graphene alone brought about a decrease in the cycle time of 27% (Fig. 4a), which is similar to the previous result^13^. In contrast, the addition of AgNFs significantly improved the cycle time by 38% at a mass ratio of AgNFs to graphene of 1 (Fig. 4b); the improvement is greater than that seen when only graphene is used to coat the TCA. This demonstrates that the AgNFs improved the effective thermal properties of the coating layer on the TCA, resulting in enhanced dynamic responses of the TCA. The maximum strain of the TCA remained almost constant at about 13.3% before and after the graphene/AgNF hybrid coating.

**Figure 5:**
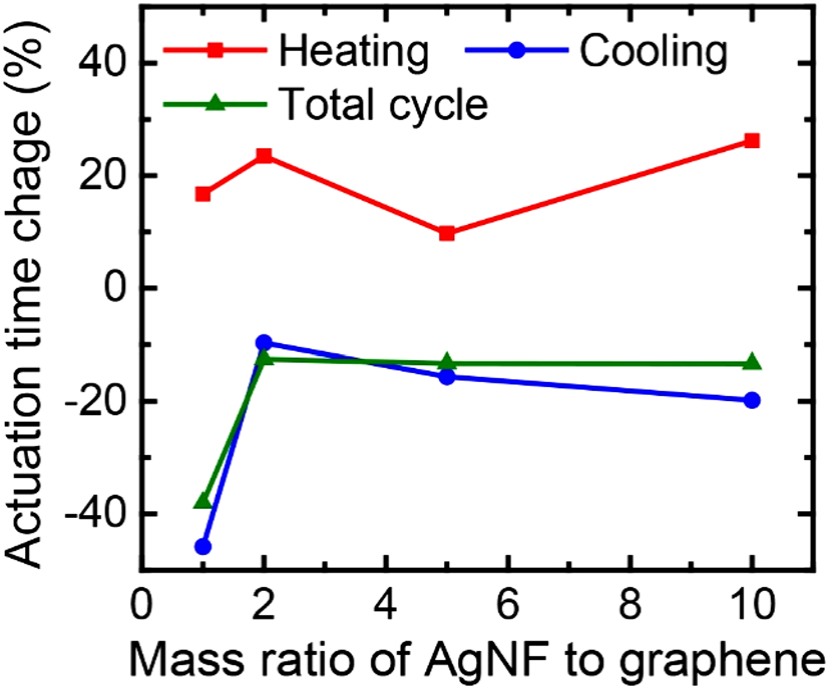
Cycle time changes of the graphene/AgNF-coated TCAs as a function of the change in mass ratio of AgNFs to graphene.

The effect of AgNF concentration on the performance of the TCAs was investigated by varying the mass ratio of AgNFs to graphene from 1 to 10. As shown in Fig. 5, it was found that an increase in the amount of AgNFs in the coating layer did not bring about much improvement in cycle time. Although more AgNFs make more thermal contacts between graphene flakes, they also increase interfacial bonding between graphene flakes. Thus, the overall stiffness of the TCA increases, which interrupts the dynamic behaviors of the TCA^13^.

**Figure 6:**
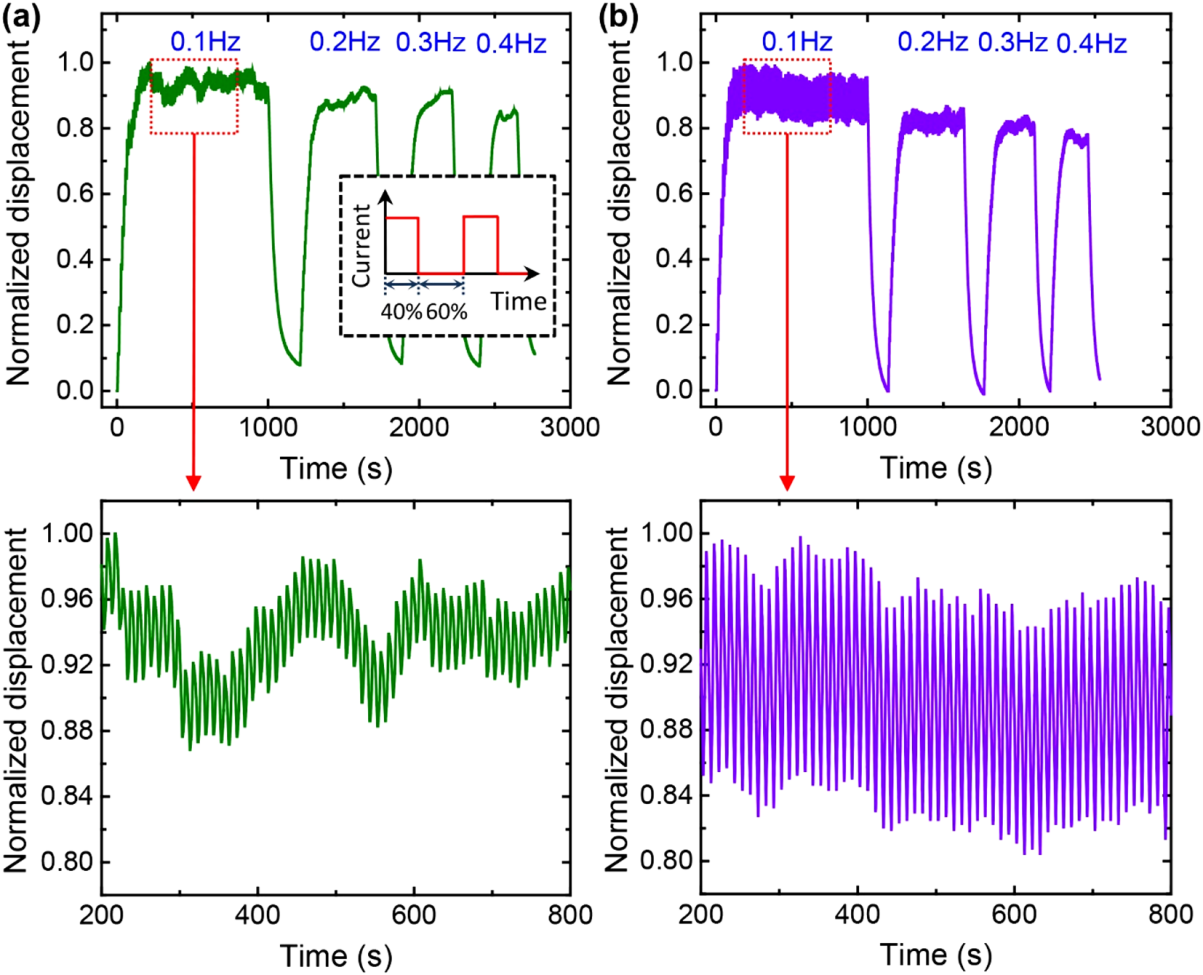
Response of the TCAs to an applied pulsed current with varying frequency. The inset shows the applied current profile with a 40% duty cycle. The frequency of the pulsed current changes from 0.1 to 0.4 Hz. (**a**,**b**) Displacement profiles normalized to the maximum displacement of the TCAs (**a**) without coating and (**b**) with graphene/AgNF coating.

In addition, the dynamic behaviors of the TCAs were characterized by monitoring the displacement responses as a function of the frequency of the heating and cooling cycles (Fig. 6). A pulsed current with a 40% duty cycle was applied to the TCA for this test. The 40% duty cycle is composed of a step current during 40% of the period, as shown in the inset of Fig. 6a. When the frequency is increased from 0.1 to 0.4 Hz, the maximum displacement of the TCA decreases because the TCA does not have sufficient time to cool. Figure 6 shows the normalized displacement profiles of TCAs with and without the coating, where the normalized displacement was calculated by dividing the measured displacements of the TCA with the maximum displacement at 0.1 Hz. Interestingly, the peak-to-peak amplitude of the oscillation in the displacements increased for the TCA coated with graphene/AgNF hybrids; the peak-to-peak amplitude for the graphene/AgNF-coated TCA is approximately 3 times larger than that for the neat TCA at a frequency of 0.1 Hz. This confirms that the graphene/AgNF coating improves the dynamic responses of the TCA by enhancing heat transfer.

## Conclusions

In summary, the dynamic behaviors of a TCA, a promising artificial muscle material, were improved by treating the surface of the actuator with a graphene/AgNF hybrid coating. A simple spray-coating method was used to obtain thin, uniform layers on the fabricated TCAs. As the nylon fibers were coated with silver, the electrically conductive TCA was actuated by Joule heating and natural cooling in air. AgNFs had ultrathin petals with tens of nanometers in thickness, which enabled coalescence of the AgNFs by heat treatment at a low temperature. Therefore, the graphene/AgNF hybrid coating dramatically reduced the total actuation time in repeated heating and cooling cycles; the maximum reduction in cycle time was 38% at a mass ratio of AgNFs to graphene of 1. This is attributable to improved interfacial thermal contacts between graphene flakes caused by coalescence of AgNFs as well as high-quality graphene flakes with extremely high in-plane thermal conductivity. Furthermore, when a pulsed current with frequencies ranging from 0.1 to 0.4 Hz was applied to the TCAs, the graphene/AgNF-coated TCA showed a threefold greater peak-to-peak amplitude in the displacement response as compared to the neat TCA without any coating. This work demonstrates the great potential of the graphene/AgNF hybrid coating for development of high-performance thermally-operated soft actuators.

## References

[1] Rus D, Tolley MT (2015). Design, fabrication and control of soft robots. Nature.

[2] Haines CS (2016). New twist on artificial muscles. Proc. Natl. Acad. Sci. U.S.A..

[3] Majidi C (2019). Soft-matter engineering for soft robotics. Adv. Mater. Technol..

[4] Kim H-I, Han M-W, Song S-H, Ahn S-H (2016). Soft morphing hand driven by SMA tendon wire. Compos. B Eng..

[5] Jani JM, Leary M, Subic A, Gibson MA (2014). A review of shape memory alloy research, applications and opportunities. Mater. Design.

[6] Martinez RV, Fish CR, Chen X, Whitesides GM (2012). Elastomeric origami: programmable paper-elastomer composites as pneumatic actuators. Adv. Funct. Mater..

[7] Jo C, Pugal D, Oh IK, Kim KJ, Asaka K (2013). Recent advances in ionic polymer-metal composite actuators and their modeling and applications. Prog. Polym. Sci..

[8] Kovacs G, During L, Michel S, Terrasi G (2009). Stacked dielectric elastomer actuator for tensile force transmission. Sens. Actuators A Phys..

[9] Haines CS (2014). Artificial muscles from fishing line and sewing thread. Science.

[10] Kim K (2018). Double helix twisted and coiled soft actuator from spandex and nylon. Adv. Eng. Mater..

[11] Yip, M. C. & Niemeyer, G. In *2015 IEEE International Conference on Robotics and Automation *(*ICRA*)*.*2313–2318.

[12] Lima MD (2012). Electrically, chemically, and photonically powered torsional and tensile actuation of hybrid carbon nanotube yarn muscles. Science.

[13] Piao C (2019). Enhanced dynamic performance of twisted and coiled soft actuators using graphene coating. Compos. B Eng..

[14] Balandin AA (2008). Superior thermal conductivity of single-layer graphene. Nano Lett..

[15] Chen SS (2011). Raman measurements of thermal transport in suspended monolayer graphene of variable sizes in vacuum and gaseous environments. ACS Nano.

[16] Lee D (2017). Dependence of the in-plane thermal conductivity of graphene on grain misorientation. Chem. Mater..

[17] Pop E, Varshney V, Roy AK (2012). Thermal properties of graphene: fundamentals and applications. MRS Bull..

[18] Sun J (2017). Preparation of boron nitride nanosheet/nanofibrillated cellulose nanocomposites with ultrahigh thermal conductivity via engineering interfacial thermal resistance. Adv. Mater. Interfaces.

[19] Muhammed Ajmal C, Faseela KP, Swati Singh, Baik S (2016). Hierarchically-structured silver nanoflowers for highly conductive metallic inks with dramatically reduced filler concentration. Sci. Rep..

[20] Ma R, Kang B, Cho S, Choi M, Baik S (2015). Extraordinarily high conductivity of stretchable fibers of polyurethane and silver nanoflowers. ACS Nano.

[21] Cho, K. H. *et al.* In *2016 6th IEEE International Conference on Biomedical Robotics and Biomechatronics *(*BioRob*)*.*94–98.

[22] Han X (2013). Scalable, printable, surfactant-free graphene ink directly from graphite. Nanotechnology.

[23] Malard LM, Pimenta MA, Dresselhaus G, Dresselhaus MS (2009). Raman spectroscopy in graphene. Phys. Rep..

[24] Chen SS (2011). Synthesis and characterization of large-area graphene and graphite films on commercial Cu–Ni alloy foils. Nano Lett..

[25] Park H, Lim S, Nguyen DD, Suk JW (2019). Electrical measurements of thermally reduced graphene oxide powders under pressure. Nanomaterials.

[26] Simeonov A (2018). Bundled super-coiled polymer artificial muscles: design, characterization, and modeling. IEEE Robot. Autom. Lett..

[27] Yip MC, Niemeyer G (2017). On the control and properties of supercoiled polymer artificial muscles. IEEE Trans. Robot..

[28] Kholmanov IN (2012). Improved electrical conductivity of graphene films integrated with metal nanowires. Nano Lett..

[29] Oluwalowo A, Nguyen N, Zhang S, Park JG, Liang R (2019). Electrical and thermal conductivity improvement of carbon nanotube and silver composites. Carbon.

[30] Huang J (2019). Silver nanoparticles decorated 3D reduced graphene oxides as hybrid filler for enhancing thermal conductivity of polystyrene composites. Compos. A Appl. Sci. Manuf..

[31] Hensel RC (2020). Monitoring the dispersion and agglomeration of silver nanoparticles in polymer thin films using localized surface plasmons and Ferrell plasmons. Appl. Phys. Lett..

